# Preserving predation

**DOI:** 10.1093/biosci/biaf121

**Published:** 2025-07-17

**Authors:** Jordan O Hampton, Thomas M Newsome, Amy Dickman, Peter Sandøe

**Affiliations:** The School of Veterinary Medicine and the Harry Butler Institute at Murdoch University, Murdoch, Western Australia, Australia; The School of Life and Environmental Sciences, University of Sydney, Camperdown, New South Wales, Australia; The Wildlife Conservation Research Unit, within the Department of Biology, Recanati-Kaplan Centre,University of Oxford, in Oxford, England, United Kingdom of Great Britain and Northern Ireland; The Department of Food and Resource Economics and the Department of Veterinary and Animal Sciences, University of Copenhagen, Frederiksberg, Denmark

**Keywords:** animal welfare, ethics, predation, veterinary, wildness

## Abstract

The One Health concept has been enthusiastically adopted in conservation. However, there are some underrecognized trade-offs that must be navigated when it comes to applying One Health ideas to wild animals. One of these relates to conflicts between the health of individual animals and the wildness and resilience of ecosystems when it comes to natural processes such as predation. Over the past few decades, there is evidence of wildlife managers providing veterinary treatment to animals injured in predation events. These efforts need to be tempered in light of concerns for biodiversity, wildness and human interests. We recognize that animals injured through predation can be distressing to contemplate for some humans, but allowing nature to take its course for these animals is enabling an essential ecological process. On this basis, we argue that there is not a strong ethical justification for interfering with predation.

Conservation has seen a recent surge in interest in wildlife diseases (Cunningham et al. 2017), encompassing emerging infectious diseases capable of causing extinctions in wildlife taxa such as amphibians (Fisher and Garner 2020), zoonotic diseases that threaten human health (Ripple et al. 2022), and noninfectious diseases such as those caused by environmental pollutants (de Jersey et al. 2023). However, the term *disease* is poorly defined and can potentially be extended to any process that causes a harmful deviation from the normal structural or functional state of an organism (Scully 2004). The focus on disease has gone hand in hand with the concept of One Health taking an increasingly influential role in conservation (Cunningham et al. 2017). One Health is an “integrated, unifying approach that aims to sustainably balance and optimize the health of people, animals and ecosystems” (WHO 2025). The One Health concept has expanded from an initial focus on transmission of parasites and infectious diseases from wildlife to humans (Atlas 2012), to a broader perspective encompassing the health of all humans, animals, and ecosystems (Cunningham et al. 2017).

The idea of One Health is inherently appealing. The paradigm adopts a win–win narrative (Crozier and Schulte‐Hostedde 2014, Jenkins et al. 2015) and provides a structured approach for *how* to develop interventions when wildlife, human, or domestic animal health is threatened (Buttke et al. 2015). However, the absence of trade-offs implied by this win–win narrative can be misleading. First, there has been a tendency to focus on human health as the only ultimate value (Sandøe et al. 2014). Second, it is not always clear what obligations are implied in the requirement to optimize the health of all animals (WHO 2025), especially when it comes to wildlife inhabiting natural ecosystems. This is especially difficult because prioritizing health often conflicts with other important values in wildlife management (Huber et al. 2011, Lynch et al. 2025). Indeed, some critical processes underpinning ecosystem functioning will unavoidably harm the health of individual animals. Examples include parasitism (Jenkins et al. 2015), competition (Hampton et al. 2019), rainfall and flooding (Wuczyński and Jakubiec 2013), forest fires (Jones et al. 2023), heat (Mo et al. 2021), cold (Mysterud et al. 2023), and even predation (Vollset et al. 2023).

It is the final example that we explore in the present article, focusing on some ethical challenges arising from the concept of predation as an issue affecting the health and welfare of wildlife that warrants intervention.

## When to intervene?

After centuries of species, populations, and ecosystems being the focus of wildlife management, there has been a recent focus on *individual* wild animals (Palmer et al. 2023). This move has coincided with interest in expanding the idea of animal welfare to encompass free-ranging animals (Beausoleil et al. 2018), culminating in the idea of One Welfare following hot on the heels of One Health (Fawcett 2020). But the ethical implications of recognizing the value of individual wild animals are not always clear. Therefore, there is room for an ethical discussion about *when* humans should intervene in the lives of wild animals and *how much* (van Dooren et al. 2023).

Historically, a central distinction in wildlife ethics has been made between human-caused (anthropogenic) processes and natural processes. While acknowledging the problematic nature of the human–nature binary in philosophy (Milstein et al. 2023), real-world wildlife interventions have historically been reserved for anthropogenic cases (Kirkwood and Sainsbury 1996), rather than natural harms (Katz 2024). Therefore, wildlife managers have broadly felt a duty to intervene when sources of suffering or disruption were anthropogenic but not when they were otherwise (Miller et al. 2023). For example, large mobilizations of human effort have occurred when oil spills affect seabirds (Piatt et al. 1990) but not when migratory ungulates drown *en masse* in rivers (Palmer 2010).

However, several recent trends in wildlife ethics have converged on the conclusion that humans *should* intervene even when wildlife are harmed by nonanthropogenic causes. These are mainly found in approaches that emphasize wild animal health (conservation medicine; Deem et al. 2001) or welfare (wild animal suffering; Horta 2017). As a natural consequence of this growing focus on health and welfare of wild animals, there has been an increasing involvement of veterinarians in wildlife management (Broz et al. 2021). This development has seen growing enthusiasm for applying the knowledge gained from domesticated and zoo animals in veterinary medicine to the health of free-ranging wildlife. This trend has seen some high-profile free-ranging wildlife species such as mountain gorillas (*Gorilla beringei beringei*) subjected to ongoing veterinary interventions, as they would in a zoo, under the rubric of conservation medicine (Cranfield et al. 2002). However, some issues may have fallen through the cracks (Cattet 2013) in this collaboration between veterinarians and wildlife managers. One of these is the separation between the technical question of what *can* be done to (or for) wild animals and the ethical question of what *ought to* be done.

## Treating predation

Over the past few decades, there is evidence of veterinarians and wildlife managers intervening in predation to treat prey and predators injured through this process (table 1). This generally entails the identification of wild animals with conspicuous visible injuries from nonlethal (i.e., unsuccessful) predation interactions, and consequent capture, anesthesia, and treatment by humans. The latter often comprises surgery or wound dressing, and administration of veterinary therapeutics, typically including antibiotics (table 2). Prior to 2000, this approach appears to have been confined to species that were often in close contact with humans and seen as charismatic (e.g., pinnipeds; Stewardson 1999). However, in the past couple of decades, this practice has been extended to more wildlife species, including terrestrial mammals such as Grévy's zebras (*Equus grevyi*) and birds such as yellow-eyed penguins (*Megadyptes antipodes*; table 1). Both of these are threatened species (IUCN 2025), which are overrepresented in the examples highlighted in table 1. It is apparent that, at present, such interventions are occurring widely but not necessarily frequently. Some organizations make public the specific treatments employed in such interventions and their frequency (table 2).

**Table 1.** Examples of reports describing the treatment of predation injuries in wild animals.

Prey species	Predator species	**Country**	**Report type**	**Citation**
Cape fur seal (*Arctocephalus pusillus pusillus*)	Multiple shark spp.	South Africa	Peer-reviewed article	Stewardson (1999)
Plains zebra (*Equus quagga*)	Unknown mammal spp.	Kenya	Online report	David Sheldrick Wildlife Trust (2007)
Grévy's zebra (*Equus grevyi*)	Lion (*Panthera leo*)	Kenya	Media article	Montanari (2017)
Bornean orangutan (*Pongo pygmaeus wurmbii*)	Sunda clouded leopard (*Neofelis diardi*)	Indonesia	Peer-reviewed article	Sunderland-Groves and colleagues (2021)
Yellow-eyed penguin (*Megadyptes antipodes*)	Unknown marine spp.	New Zealand	Conference paper	Johnson and colleagues (2022)
Black rhino (*Diceros bicornis*)	Lion	Kenya	Social media post	David Sheldrick Wildlife Trust (2024)

*Note:* The reports are listed chronologically.

**Table 2.** Examples of predation injuries treated by the David Sheldrick Wildlife Trust and Kenya Wildlife Service from January to July 2024.

**Month**	**Incident**	**Predator or prey treated**	**IUCN category of treated species**	**Treatment given**
July 2024	Lion (*Panthera leo*) injured by unknown prey species	Predator	Vulnerable	Capture, anaesthesia, wound cleaning, antibiotic, nonsteroidal anti-inflammatory, anthelmintic, insecticide
July 2024	Lion injured by unknown prey species	Predator	Vulnerable	Capture, anaesthesia, wound cleaning, antibiotic, anthelmintic
June 2024	Plains zebra (*Equus quagga*) injured by lion	Prey	Near Threatened	Capture, anaesthesia, wound cleaning, antibiotic, steroidal anti-inflammatory, anthelmintic
June 2024	Plains zebra injured, likely by predators, and a snare	Prey	Near Threatened	Capture, anaesthesia, wound cleaning, antibiotic, nonsteroidal anti-inflammatory
June 2024	Black rhino (*Diceros bicornis*) attacked by lions	Prey	Critically endangered	Capture, anaesthesia, wound cleaning, antibiotic, nonsteroidal anti-inflammatory
April 2024	Lion injured by unknown prey species (antelope)	Predator	Vulnerable	Capture, anaesthesia, wound cleaning, antibiotic, steroidal anti-inflammatory, anthelmintic
January 2024	Black rhino attacked by lions	Prey	Critically endangered	Capture, anaesthesia, wound cleaning, antibiotic, nonsteroidal anti-inflammatory
January 2024	Black rhino attacked by lions	Prey	Critically endangered	Capture, anaesthesia, wound cleaning, antibiotic, nonsteroidal anti-inflammatory
January 2024	Plains zebra injured by unknown predator species	Prey	Near threatened	Capture, anaesthesia, wound cleaning, antibiotic, nonsteroidal anti-inflammatory, anthelmintic

*Source:* Adapted from The David Sheldrick Wildlife Trust (2024).

## Unintended consequences

From the One Health perspective of optimizing the health of all animals, veterinary treatment to mitigate the effects of predation may seem to make good sense. Particularly so if there are clear negative animal welfare impacts occurring and if humans have the resources to treat the affected animals. Even from the point of view of more mainstream ecocentric positions, where protection of biodiversity or wildness are overriding concerns (Palmer et al. 2023), such treatment may appear unnecessary but still innocuous because of its limited scale. That is, unless the idea were to gain traction and wildlife managers felt public pressure to treat (or prevent) predation at a broad scale, and it became the accepted norm.

It is therefore worth pausing to consider what long-term consequences might be anticipated from a global move toward treating predation. Key questions include the following. Which species deserve intervention and why; for example, should it only apply to critically endangered species (see table 2)? Or only native species? Or only species in close proximity to humans? Or only species for which sentience is broadly recognized? Or only under circumstances where humans have disrupted historical predator–prey dynamics? This final consideration relates to the concept of anthropogenic versus natural processes and the difficulty in differentiating the two in the Anthropocene (Paula Vieira and Anthony 2021). We don't perceive any obvious answers to these questions at present. And if these interventions became much more widespread, what would be the likely effects on populations of wild predators and scavengers, particularly taxa such as vultures which rely on carrion and are often highly imperiled (Ives et al. 2022)? What impacts would it have on natural selection (Crozier and Schulte‐Hostedde 2014)? And finally, what sort of ecosystems would we be left with if such practices became widespread or even *universal*?

Some tentative answers can be offered in the present article (figure 1). For example, the central role of predation in regulating ecosystems and ecological processes could be impaired, including the role of carrion in maintaining ecosystem stability (Wilson and Wolkovich 2011). Wild predators (Ripple et al. 2014) and scavengers (Buechley and Şekercioğlu 2016) are already disproportionately impacted by the extinction crisis. Losing access to wounded prey animals (and their carcasses) would have detrimental impacts on the welfare of such animals and their populations. For some predator species, this would be particularly significant—for example, Komodo dragons (*Varanus komodoensis*), whose predatory ecology relies on the effects of venom and hemorrhage to kill their prey animals relatively slowly (Fry et al. 2009). Scavengers also play a major role in preventing transmission of infectious diseases from carcasses, including to humans (Markandya et al. 2008), so a loss of food resources and subsequent decline of scavengers would impact this ecosystem service. Natural selection pressure from coevolving with predators is what endowed many prey species with athleticism, horns, or antlers. Removal of this selection pressure would significantly change the course of evolution for these species (Deem et al. 2001). In an unlikely world in which predation intervention became *universal*, food webs would likely collapse from major negative impacts on carnivores (Ripple et al. 2014).

**Figure 1.**
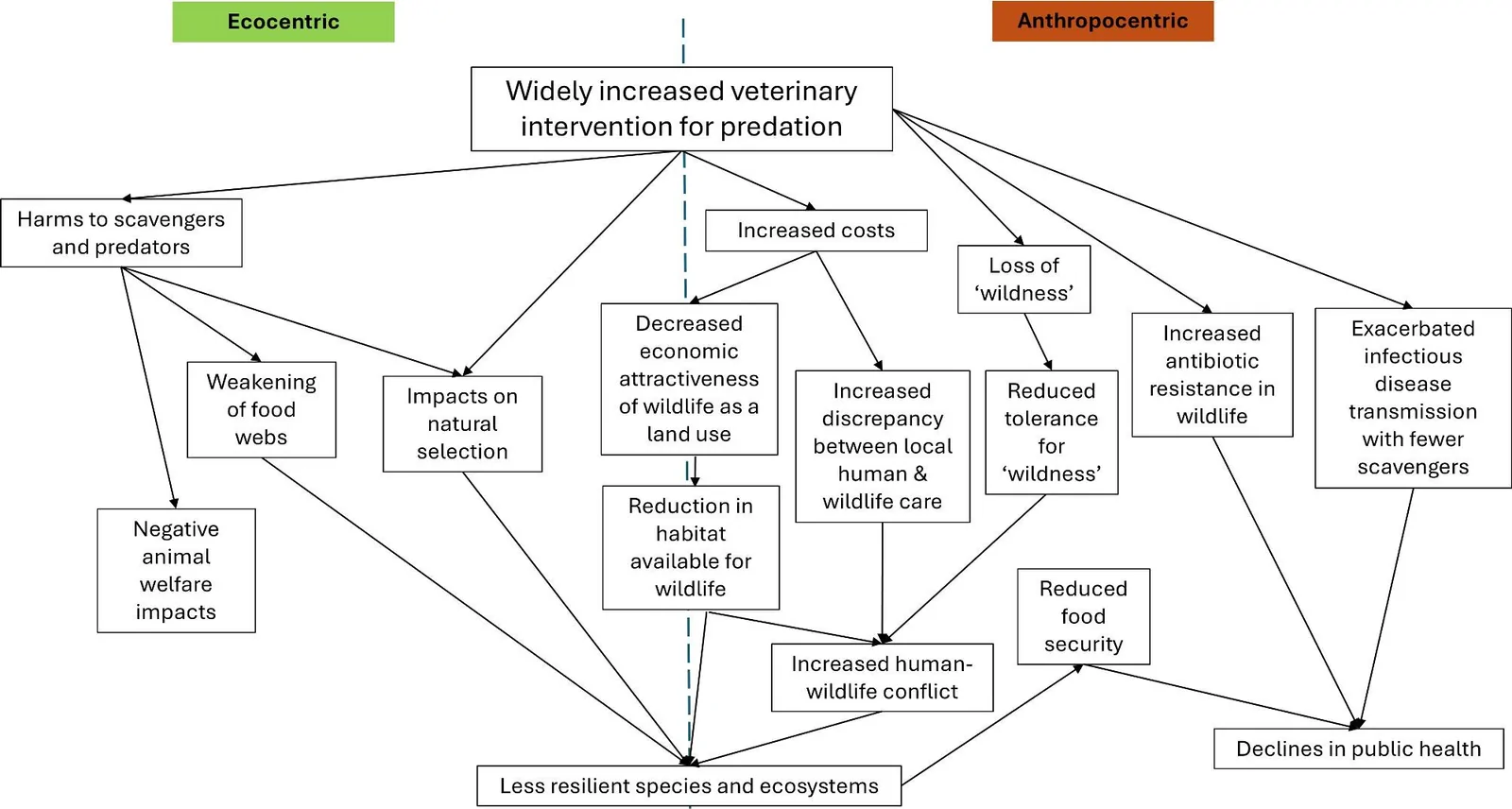
Some of the likely disvalues that would be incurred if interventions to treat predation were to become more widespread.

Beyond these ecological effects, it is worth considering the human implications for future management of wild animals if the idea were to become mainstream that predation is a health issue that requires intervention. First, what would it *cost* to police ecosystems in this way? And what would be the opportunity cost? Given the general overlap between biodiversity and poverty (Fisher Brendan and Christopher 2007), wildlife provided with expensive veterinary treatments may be afforded standards of care that exceed those available in human healthcare in some developing countries. This prospect raises justice concerns (Tan 2021) that are likely to intensify human–wildlife conflict (figure 1), one of the major emerging global conservation threats.

Not only would such an approach to wildlife management come at enormous ecological and financial cost, but it would also come at the expense of the wildness of these animals (Lorimer and Driessen 2014), relating to the idea of wild animals shaping their own evolution through agency. In a nutshell, we can choose to keep wildlife wild or safe (Pride Lion Conservation Alliance 2021), but they cannot be both. Indeed, the recent phenomena of treating predation might be another symptom of growing paternalism in conservation (Palmer 2021). These efforts have also been described as manifestations of pressure to sanitize the wild (Pride Lion Conservation Alliance 2021). This trend has been suggested to represent a slow transformation from *in situ* conservation to a form of free-range zookeeping or simply turning nature into a zoo (Johannsen 2017).

Finally, growing intolerance of wildlife predation may undermine the social license of other wildlife management initiatives that rely on the process of predation to achieve ecological goals. If the idea became entrenched that predation is an undesirable threat to animal health and welfare, any efforts to reintroduce predators to landscapes and seascapes from which they had been extirpated (Ritchie et al. 2012), under the framing of rewilding (or ecological restoration; Finnerty et al. 2025), or even deextinction (Jørgensen 2013) could be imperiled (Allen et al. 2019). Indeed, calls to prevent such initiatives are already being made on these grounds (Horta 2010, Katz 2024).

## Veterinarization

The trend toward treating wildlife as veterinary patients, referred to as *veterinarization* (Broz et al. 2021) might also set the scene for expansion of other (similar) types of health-related interventions. These include feeding of wildlife or other forms of provisioning (Cox and Gaston 2018), treatment of parasitism using anthelmintic chemicals (Eleftheriou 2021), or treatment of infectious diseases using antibiotics (Vlčková et al. 2016). The use of antibiotics in wild animals, which is seemingly common in cases where predation injuries are treated (table 2) raises a separate set of public health concerns relating to the development of antimicrobial resistance (Swift et al. 2019), see figure 1.

## Where to now?

Maybe veterinary treatment of predation *won't* become much more widespread. But then again, maybe it *will*, and it is already becoming more expected in certain locations, such as in parts of Kenya (table 2). It is noteworthy that these interventions are currently being applied to some species that are not critically endangered, such as the plains zebra (*Equus quagga*), listed as Near Threatened (IUCN 2025). In the age of One Health, this may become a moral slippery slope, in which concept creep (Haslam 2016) leads to more and more manifestations of predation being targeted for intervention. This is, after all, exactly what has happened when ethicists have taken the idea of predation as a moral evil (Torres 2015) to its most extreme logical conclusion, culminating in efforts to herbivorize predators (Herbivorize predators 2024) or even calls for the intentional eradication of all wildlife predators (Bramble 2021).

Even assuming those extremes are never realized, we see a need for an ethical discussion of the trend toward increasing intervention in the lives of wild animals by means of veterinary medicine. We suggest that this specific context (limited in scope) fits within a broader need to rethink ethical guidelines when it comes to wildlife and intervention in the Anthropocene (Paula Vieira and Anthony 2021). If wildlife managers fail to reevaluate their criteria for intervention in the lives of wild animals, they may find themselves pressured to intervene in other aspects of wild animals’ lives. However, for those who, like the authors of this article, see conservation of wild biodiversity as a key value alongside animal health and welfare, there is a need to highlight the negative effects on wild biodiversity of unnecessary veterinary interventions.

We feel that the global wildlife management community needs to proactively have a conversation about where ethical commitments lie—and where they don't. We recognize that predation causes pain and death and may be distressing to witness for some humans (Clauss et al. 2025). But it is a natural and essential ecological process, and any justification to intervene in this process must address unavoidable trade-offs with biodiversity and wildness. Failing to address these trade-offs has been described as an approach that prioritizes visible costs but risks major hidden harms (Pride Lion Conservation Alliance 2021). If these concerns are not given weight in the One Health era, we might find that the wildlife we are left with are less wild and inhabiting ecosystems that are less diverse and less resilient.

## Author Biography

Jordan O. Hampton (jordan.hampton@murdoch.edu.au) is affiliated with the School of Veterinary Medicine and the Harry Butler Institute at Murdoch University, in Murdoch, Western Australia, in Australia. Thomas M. Newsome is affiliated with the School of Life and Environmental Sciences at the University of Sydney, in Camperdown, New South Wales, in Australia. Amy Dickman is affiliated with the Wildlife Conservation Research Unit, within the Department of Biology, at the Recanati-Kaplan Centre, at the University of Oxford, in Oxford, England, in the United Kingdom of Great Britain and Northern Ireland. Peter Sandøe is affiliated with the Department of Food and Resource Economics and the Department of Veterinary and Animal Sciences at the University of Copenhagen, in Frederiksberg, Denmark.
